# Follow-up in a point-of-care diabetic retinopathy program in Pittsburgh: a non-concurrent retrospective cohort study

**DOI:** 10.1186/s12886-024-03581-9

**Published:** 2024-08-20

**Authors:** Francisco J. Bonilla-Escobar, Maria Regina Eibel, Laura Le, Denise S. Gallagher, Evan L. Waxman

**Affiliations:** 1 Department of Ophthalmology, University of Pittsburgh, UPMC Vision Institute, Pittsburgh, PA USA; 2 Grupo de Investigación Visión y Salud Ocular, Servicio de Oftalmología, Universidad del Valle, Hospital Universitario del Valle, Cali, Colombia; 3 Fundación Somos Ciencia al Servicio de la Comunidad, Fundación SCISCO /, Science to Serve the Community Foundation, SCISCO Foundation, Cali, Colombia

**Keywords:** Diabetic Retinopathy Examination, Screening Program, Follow-up, Point-of-care, Ocular Findings

## Abstract

**Background:**

The Point-of-Care Diabetic Retinopathy Examination Program (POCDREP) was initiated in 2015 at the University of Pittsburgh/UPMC in response to low diabetic retinopathy (DR) examination rates, a condition affecting a quarter of people with diabetes mellitus (PwDM) and leading to blindness. Early detection and treatment are critical with DR prevalence projected to triple by 2050. Approximately, half of PwDM in the U.S. undergo yearly examinations, and there are reported varying follow-up rates with eye care professionals, with limited data on the factors influencing these trends. POCDREP aimed to address screening and follow-up gap, partnering with diverse healthcare entities, including primary care sites, free clinics, and federally qualified health centers.

**Methods:**

A non-concurrent retrospective cohort study spanning 2015–2018 examined data using electronic health records of patients who underwent retinal imaging. Imaging was performed using 31 cameras across various settings, with results interpreted by ophthalmologists. Follow-up recommendations were made for cases with vision-threatening DR (VTDR), incidental findings, or indeterminate results. Factors influencing follow-up were analyzed, including demographic, clinical, and imaging-related variables. We assessed the findings at follow-up of patients with indeterminate results.

**Results:**

Out of 7,733 examinations (6,242 patients), 32.25% were recommended for follow-up. Among these, 5.57% were classified as having VTDR, 14.34% had other ocular findings such as suspected glaucoma and age-related macular degeneration (AMD), and 12.13% were indeterminate. Of those recommended for follow-up, only 30.87% were assessed by eye care within six months. Older age, marriage, and severe DR were associated with higher odds of following up. Almost two thirds (64.35%) of the patients with indeterminate exams were found with a vision-threatening disease at follow-up.

**Conclusion:**

The six-month follow-up rate was found to be suboptimal. Influential factors for follow-up included age, marital status, and the severity of diabetic retinopathy (DR). While the program successfully identified a range of ocular conditions, screening initiatives must extend beyond mere disease detection. Ensuring patient follow-up is crucial to DR preventing programs mission. Recommended strategies to improve follow-up adherence include education, incentives, and personalized interventions. Additional research is necessary to pinpoint modifiable factors that impact adherence and to develop targeted interventions.

## Background

Diabetic Retinopathy (DR) affects a quarter of people with diabetes mellitus (PwDM) and is the leading cause of blindness among working-age adults in the United States [1, 2]. With the worsening diabetes epidemic, the prevalence of DR is projected to triple from 2005 levels to affect 16 million Americans by the year 2050 [3]. Blinding complications from DR can be prevented with early detection and treatment [4–7]. Nevertheless, fewer than half of the PwDM in the United States are examined yearly [8–11].

In 2015, it was stablished the Point-of-Care Diabetic Retinopathy Examination Program (POCDREP) within the University of Pittsburgh/UPMC, to address low DR exam (DRE) rates in Western Pennsylvania. The program, based on the Ophthalmology Department at the University of Pittsburgh/UPMC, took over a previous experience in the city and has partnered with primary care and endocrine sites (part of UPMC Community Medicine Inc., CMI or UPMC University of Pittsburgh Physicians, UPP), and a healthcare gap closure initiative within UPMC. The POCDREP has also partnered with Federally Qualified Health Centers (FQHCs), and local free clinics which makes it a unique program. [12, 13].

The prevention of DR does not end with a DRE; an in-person exam with an ophthalmologist after a follow-up recommendation is an essential part of the whole process. In-person evaluations are due to positive result of vision-threatening DR (VTDR), incidental findings (i.e., age-related macular degeneration [AMD], glaucoma suspicion, etc.) or indeterminate (non-gradable images) results. Incidental findings within DRE appear in about the same or higher proportions than DR (around 10–15% of the exams) [14–24]. On the other side, indeterminate results are observed in less than 10% of exams [14–24]. These results stem from ocular component opacities (cornea, lens, vitreous) or small pupils. Small pupils may be physiological or result from autonomic neuropathy, leading to reduced retinal exposure to light [21, 23, 25, 26]. Therefore, we hypothesize that PwDM with indeterminate results may have underlying pathological findings.

In our experience, one-third of POCDREP patients are recommended to follow up, similar to what is described in other programs [14–24]; however, follow-up rates range from 10 to 62% [27–38]. Information about associated factors with follow-up after DRE is limited [29–33]. Described associations include (1) non-modifiable factors, such as sex [35], older age, and non-Hispanic ethnicity [34]; (2) socioeconomic factors such as distance to eye centers [36], insurance coverage [34], and rural residency [35]; (3) self-efficacy indicators such as awareness of HbA1c levels, and capacity to schedule appointments [30]; (4) severity of DR [34]; and (5) the use of artificial intelligence for DRE [35]. Furthermore, to the best of our knowledge, there is no available information about real-life findings after indeterminate DREs and no US programs are implemented across different healthcare institutions, making the POCDREP a unique setting to identify real-life DRE outcomes.

This study aims to describe the POCDREP causes for referrals to in-person exams, follow-up rates for people referred for further evaluation, ocular findings of in-person evaluations for participants with indeterminate exams, and associated factors with follow-up. Through this research, we seek to contribute insights into the issue of adherence to recommendations in primary eye care, steering towards the improvement of program outcomes.

## Methods

### Study design

We conducted a non-concurrent retrospective cohort study to identify adherence to follow-up recommendations after DRE of PwDM in the POCDREP, factors associated with follow-up, and findings in indeterminate exams. We used data generated by automated reports from UPMC Electronic Health Record (EHR) system (Epic®) from 2015 to 2018. Participating sites in the POCDREP are UPMC-affiliated, FQHCs, and free care clinics. The University of Pittsburgh institutional review board waived the requirement for obtaining patient consent and approved reporting the results from the program (code: STUDY19120237), in accordance with the principles of the Declaration of Helsinki.

### Study participants

Patients were eligible for imaging if they were 18 years or older, were diagnosed with any diabetes mellitus type, and had been imaged between 2015 and 2018. The cutoff for age was defined because individuals with diabetes younger than 18 are less likely to develop changes in the retina [39]. We excluded repeated DREs within a year of examination in which the findings did not differ from the first exam (*n* = 75). Participants could have more than one exam in the study period.

### Procedures and settings

The number of cameras in the program at the time of the study was 31. They were shared by 59 primary care clinics, endocrine offices, and a mobile healthcare initiative offering various screening tests, including retinal photography. The clinic staff obtained images using either the Centervue DRS or Topcon TRC NW400 digital retinal camera, configured to capture a macula-centered, 45-degree image of each eye. Images from UPMC offices were stored and forwarded using Epic, while images from non-UPMC facilities were forwarded via secure e-mail. They were then reviewed and reported on by two practicing ophthalmologists. Standardized reports were created and transmitted to ordering physicians via the EHR or secure email.

### Study variables

We collected data at the individual and eye level. Individual data included demographic and clinical variables at the time of imaging from automatic monthly data reports from Epic. Demographic variables included age, sex, race (Black, White, other), ethnicity, ZIP code, patient-reported language, marital status (married for those in committed relationships or who are legally married, and unmarried for those divorced, separated, single, unknown or widowed), and insurance carrier. We recategorized insurance carriers into insurance types (I.e., Medicaid, Medicare, Dual, Commercial, Veteran’s Affairs [VA], and uninsured) and in a dichotomized variable with commercial vs. all others. ZIP codes were used as a surrogate indicator for socioeconomic status, as described previously [12]. Clinical variables included hemoglobin A1c levels (HbA1c), body mass index (BMI), BMI in categories (normal including underweight, overweight BMI = 25–29.9 kg/m^2^, obesity class I BMI = 30–34.9, obesity class II BMI = 35–39.9, obesity class III BMI ≥ 40), and smoking status at the time of imaging (never smoked, former smoker, and smoker including current everyday smoker, current occasional smoker, heavy and light tobacco smoker, and passive smoker).

Data at the eye level involved variables about the exam, including image quality, diagnosis, and recommendation. Image quality was recorded per eye and classified as good, fair, just gradable, poor, non-gradable, or no image. Good quality images included all the relevant parts of the retina to be examined for DR (macula, disc, temporal vascular branches, and near retinal periphery) without significant artifacts or shadows. Fair-quality images had a shadow or an artifact but were considered easily interpretable. Images described as ‘just gradable’ had multiple artifacts (i.e., eyelashes, shadows, mild blur) but were considered sufficient to rule out DR. Images were described as ‘poor’ if the image quality would not have been sufficient to rule out DR, but nevertheless demonstrated abnormal findings. An indeterminate exam was defined as non-gradable or no image in either of the examined eyes. We described the quality of image per eye and per person (both eyes).

Patients with no retinopathy or mild non-proliferative DR (NPDR) were recommended to have a new exam in a year. Findings of moderate NPDR, severe NPDR, proliferative DR, or macular exudate (a surrogate marker of clinically significant diabetic macular edema, DME) [40–42] were classified as VTDR. Patients with VTDR, other vision-threatening findings (glaucoma suspicion, AMD, etc.), or indeterminate examinations (non-gradable images or an eye not imaged) were recommended to follow up with an ophthalmologist for further evaluation.

EHRs were reviewed to identify follow-up and date of encounters for patients referred for further evaluation. Evidence of follow-up could take the form of (1) documentation of a subsequent eye exam in Epic; (2) eye exam reports scanned into Epic; and (3) physician notes that addressed the results of a DRE, not otherwise documented in Epic. A subsequent appointment was considered a follow-up if an ophthalmologist saw the patient within six months of imaging (≤ 180 days). Through this approach, we collected the ophthalmological findings per participant at follow-up for indeterminate eye exams but widening the timeframe up to a year post-DRE.

With the collected dates (date of exam, date of report, date of follow-up), we calculated the days from exam to report and days from report to follow-up. Days from exam to report were recategorized in DRE results produced the same day, in one (1) day, two to three (2-3) days, four to six (4-6) days, and seven (7) or more days.

### Statistical analysis

#### Univariate and bivariate analysis

Data was split into three subsets: (1) participants/individuals (*n* = 6,242), (2) exams (*n* = 7,733), and (3) exams with a follow-up recommendation (*n* = 2,494). We performed analyses at both individual and eye levels. Individual-level analyses included demographic and clinical variables, while eye-level analyses focused on image quality and specific retinal findings. Descriptive statistics were carried out using central tendency and dispersion measurements (interquartile range: IQR; standard deviation: SD) for continuous variables and frequencies and percentages for categorical variables. Missing data was counted as a separate category. As the data came from automatic data extraction, there was no additional information to be identified within the medical record to address missing values.

Image quality was recategorized per eye as gradable (good, fair, just gradable, poor quality) or non-gradable (non-gradable or no image) and compared using McNemar’s test for correlated data [43] to identify if there was one eye with a higher percentage of non-gradable images.

We compared the days from the report to follow-up based on exam results (VTDR, other diagnoses, or indeterminate exam) using the Kruskall-Wallis test, hypothesizing that people will follow up earlier or later based on the diagnosis received.

Bivariate analyses were conducted to examine associations among the individuals’ characteristics and follow-up status (not followed up vs. followed up). T-tests or Wilcoxon tests were used to examine associations between quantitative variables and follow-up status; the Chi2 test was used to examine associations among categorical variables (i.e., sex, race, etc.) with follow-up status.

#### Multivariate analysis

We built a multivariate model to evaluate associated factors with follow-up after a positive or indeterminate DRE. We carried out a two-step process to select the model’s independent variables. The first step was selecting from the study variables those described in the literature as associated with follow-up. We included insurance coverage, severity of retinopathy [34], age [30, 35], and sex [35]. The second step to select independent variables for the model via simple logistic regressions clustered by imaging site, using each study variable as a covariate in the models. With these regressions, we explored other variables from our dataset as potential confounders. Variables associated with follow-up with a *p*-value < 0.20 were included in the multivariate model [44].

We had two clustering variables: individuals and imaging sites. Patients represented one cluster of exams, as we had patients with more than one DRE in the study period. Imaging sites represented another cluster, as exams happened in different locations that grouped patients. We used a multilevel mixed-effects logistic regression to account for clusters.

Using the likelihood-ratio test, we tested the need for a multilevel multivariate model using the full model, with both clusters compared to a model without clusters. The model with two clusters was significant (*p* < 0.0001); therefore, both were kept in the final model. The variance explained by the clusters (DRE site and patient) was identified with the residual intraclass correlation coefficient (ICC).

Statistical significance was set at *p*-value < 0.05, and all the analyses were carried out using Stata 18 (StataCorp, TX)®.

## Results

### Participants/individuals level (*n* = 6,242)

We included a total of 7,733 examinations of 6,242 individuals. The mean participant age at the first exam was 57 ± 13 years; 47.31% (2,953) were female, and 55.59% (3,470) were single. People identified themselves mostly as White (59.53%, 3,716) or Black (33.56%, 2,095), and only 1.46% (91) considered themselves Latino. Most participants speak English (95.58%, 5,966), and most had type-2 diabetes (87.60%, 5,468, Table 1). There were 81.85% (5,109) people with a single exam in the study period, 13.22% (825) with two exams, 4.20% (262) with three, and 0.74% (46) with four exams.

**Table 1:** Participants’ characteristics at first examination in the Point-of-Care Diabetic Retinopathy Examination Program (POCDREP, *n* = 6,242)

Characteristics	
Age, mean (SD)^a^	57 (13.47)
Age groups, n (%)	
18–45	1,161 (18.60)
46–55	1,461 (23.41)
56–65	1,943 (31.13)
66–75	1,178 (18.87)
76–97	468 (7.50)
* Missing*	31 (0.50)
Sex, n (%)	
Female	2,953 (47.31)
Male	3,289 (52.69)
Race, n (%)	
Black	2,095 (33.56)
White	3,716 (59.53)
Others	431 (6.90)
Latino, n (%)	91 (1.46)
Marital status, n (%)	
Single	3,470 (55.59)
Married	2,555 (40.93)
* Missing*	217 (3.48%)
Type of diabetes, n (%)	
Type 1	252 (4.04)
Type 2	5,468 (87.60)
* Missing*	522 (8.35)

*Legend: SD* Standard deviation. Missing values are counted as their own category

^a^Age has 31 (0.50%) missing values

#### Exams (*n* = 7,733)

At the time of examination, participants lived in 293 different boroughs in Pennsylvania. There were no people living in areas of extreme poverty, but 10.03% (776) lived in an area of some poverty (Q3-Q4) when examined. In terms of insurance type, 61.03% (4,719) were enrolled in state/federal-assisted insurance plans (Medicare, Medicaid, dual, or VA coverage), while 30.95% (2,393) were enrolled in commercial insurance plans, and 1.90% (147) were uninsured (Table 2).

**Table 2:** Characteristics of participants at examination in the Point-of-Care Diabetic Retinopathy Examination Program (POCDREP, *n* = 7,733)

**Characteristics**	
Quartile of poverty, n (%)	
< 10% (Q1)	4,141 (53.55)
10–19.9% (Q2)	2,669 (34.51)
20–29.9% (Q3)	438 (5.66)
30–39.9% (Q4)	338 (4.37)
* Missing*	147 (1.90)
Insurance type, n (%)	
Commercial	2,393 (30.95)
Medicaid	1,479 (19.13)
Medicare	2,505 (32.39)
Dual	698 (9.03)
Veterans Affairs	37 (0.48)
Uninsured	147 (1.90)
* Missing*	474 (6.13)
BMI, mean (SD)^a^	33.73 (8.04)
BMI categories, n (%)	
Underweight (BMI < 18.5)	39 (0.5)
Normal (BMI = 18.5–24.9)	811 (10.49)
Overweight (BMI = 25–29.9)	1,822 (23.56)
Obesity class I (BMI = 30–34.9)	2,035 (26.32)
Obesity class II (BMI = 35–39.9)	1,424 (18.41)
Obesity class III (BMI ≥ 40)	1,478 (19.11)
* Missing*	124 (1.60)
HbA1C, mean (SD)^a^	7.80 (1.98)
Smoking status, n (%)	
Former smoker	2,567 (33.2)
Never smoked	3,109 (40.2)
Current smoker	1,836 (23.74)
* Missing*	221 (2.85)

*Legend: SD* Standard deviation, *BMI *Body mass index, *HbA1C* Glycated hemoglobin. Missing values are counted as their own category

^a^BMI and HbA1c had 1.60% (124) and 66.86% (6,717) of missing values, respectively

Participants’ mean BMI and HbA1c nearest to the examination time were 33.73 ± 8.04 kg/m^2^ and 7.80% ± 1.98, respectively. Based on BMI, 87.41% (6,759) of participants were overweight or above this category. A third of the participants were former smokers (33.19%, 2,567), 40.20% (3,109) had never smoked, and 23.74% (1,836) were active smokers (Table 2), with 19.06% (1,474) smoking every day.

Most examinations occurred at primary care practices (94.19%, 7,283); most were UPMC-associated practices (91.06%, 7,042). The other 4.09% (316) were at FQHCs, 2.26% (175) at free-care clinics, and 2.59% (200) with the mobile camera (Table 3). While 727 primary care providers ordered a DRE, a minority (106, 14.6%) were responsible for ordering half of the exams.

**Table 3:** Exams’ characteristics in the Point-of-Care Diabetic Retinopathy Examination Program (POCDREP, *n* = 7,733)

**Characteristic**	
Clinic administration, n (%)	
UPMC Community Medicine Inc. (CMI)	3,839 (49.64)
UPMC University of Pittsburgh Physicians (UPP)	3,203 (41.42)
Federally Qualified Health Centers	316 (4.09)
Free clinics	175 (2.26)
Mobile unit—UPMC Health Plan	200 (2.59)
Specialty, n (%)	
Family medicine	3,622 (46.84)
Internal medicine	3,434 (44.41)
Family and internal medicine	227 (2.94)
Endocrinology	249 (3.22)
* Missing*	201 (2.60)
Time from exam to report, n (%)	
0 days	6,151 (79.54)
1 day	297 (3.84)
2 days	192 (2.48)
3 days	134 (1.73)
4 days	96 (1.24)
5 days	60 (0.78)
6 days	93 (1.20)
7 days	89 (1.15)
≥ 8 days	419 (5.42)
* Missing*	202 (2.61)

*Legend*: *UPMC* University of Pittsburgh medical center

At the eye level, 88.93% (6,877) of the tests were classified as gradable. We found that 56.27% (4,351) exams had good-quality photos in both eyes, and only 6.5% (503) had both eyes with either non-gradable or no images. There was a significantly higher proportion of non-gradable images in the left eyes (10.81%, 836) when compared with the right eyes (8.55%, 661; McNemar’s chi2 test, *p* < 0.0001).

**Fig. 1:**
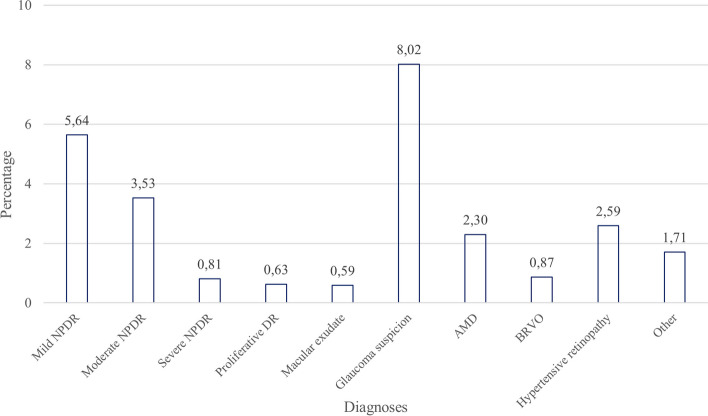
Percentage of diagnoses in the Point-of-Care Diabetic Retinopathy Examination Program (POCDREP), 2015–2018 (*n* = 7,733). *Legend:* We describe the most severe level of diabetic retinopathy found per exam (both eyes). An exam can have DR and other findings, such as suspected glaucoma, etc. NPDR: Non-proliferative diabetic retinopathy; AMD: Age-related macular degeneration; BRVO: Branch retinal vein occlusion

Interpretation of the exam was mostly carried out on the same day of imaging (79.54%, 6,151), with a minority (5.42%, 419) being interpreted after more than a week (≥ 8 days, Table 3). The exams were classified as positive for DR in 11.21% (867), negative in 76.66% (5,928), and indeterminate in 12.13% (938) of the exams. VTDR was found in 5.57% (431) of the exams (moderate NPDR 3.53%, 273; severe NPDR 0.81%, 63; proliferative DR 0.63%, 49; macular exudate, ME, 0.59%, 46, Fig. 1).

Suspicion or findings of diseases other than DR were found in 14.34% (1,109) of the total exams. Incidental findings, where more than one could be found per person, included ‘suspected’ glaucoma (8.02%, 620), hypertensive retinopathy (2.59%, 200), AMD (2.30%, 178), and Branch Retinal Vein Occlusion (BRVO, 0.87%, 67). Other abnormal findings included epiretinal membranes, choroidal nevus, asteroid hyalosis, and chorioretinal and macular scars (1.71%, 132, Fig. 1).

Approximately one-third (32.25%, 2,494) of DREs resulted in a recommendation for an in-person follow-up due to an indeterminate result, the identification of vision-threatening findings, or other abnormal findings.

### Follow-up required (*n* = 2,494)

For those recommended for follow-up, eye care visits could be confirmed in the next six months from the report date in 30.87% (770) of the patients. About half of the follow-up exams were performed with UPMC eyecare providers (57.92%, 446). The median time in days to follow-up from interpretation was 55 (IQR = 28–98). A shorter time to follow-up after interpretation was observed in participants diagnosed with BRVO and VTDR, while the longest time was observed in those with indeterminate exams (Kruskall-Wallis test *p* = 0.005, Table 4).

**Table 4:** Days to follow-up after retinal exam findings in POCDREP (*n* = 770)

Condition	n	Median (IQR)	Mean (SD)
All that followed-up	770	55 (28–98)	66.48 (48.39)
VTDR	182	46.5 (23–84)	58.92 (47.18)
Indeterminate exam	210	69 (29–114)	76.3 (51.16)
Glaucoma suspect	228	54.5 (29–90)	64.29 (46.2)
AMD	45	50 (22–98)	65.2 (47.64)
Hypertensive retinopathy	28	55.5 (18–95)	66 (55)
BRVO	28	38 (15–110)	57.18 (51.37)
Other conditions	36	48 (17.5–84.5)	59.31 (47.12)

*Legend*: *POCDREP* Point-of-care diabetic retinopathy examination program, *IQR* Interquartile rank, *SD* Standard deviation, *VTDR* Vision-threatening diabetic retinopathy, *AMD* Age-related macular generation, *BRVO* Branch retinal vein occlusion

For patients recommended to follow-up for VTDR, less than half (42.23%, 182/431) had a documented follow-up visit within six months of DRE. Follow-up for BRVO was 46.67% (28), 37.50% (228) for suspected glaucoma, 31.03% (45) for AMD, 29.47% (28) for hypertensive retinopathy, and 35.64% (36) for other diagnoses. The follow-through rate for patients with indeterminate exams was 24.68% (210) in the next six months of DRE.

A third (33.80%, 317) of patients with indeterminate exams had followed up one year after DRE. Nearly two-thirds (64.35%) of these had findings that had gone undetected in DRE, and 12.62% had VTDR (Table 5).

**Table 5:** Findings at follow-up examinations for patients with indeterminate diabetic retinopathy exams (*n* = 317)

Findings	n (%)
Within normal limits	113 (35.65)
Diabetic retinopathy	
Mild NPDR	33 (10.41)
Moderate NPDR	14 (4.42)
Severe NPDR	3 (0.95)
Proliferative DR	13 (4.10)
Macular Edema	9 (2.84)
Panretinal photocoagulation	1 (10.32)
Other vision-threatening conditions	
Glaucoma	42 (13.25)
Age-related macular degeneration	8 (2.52)
Hypertensive retinopathy	9 (2.84)
Branch retinal vein occlusion	4 (1.26)
Cataract	115 (36.28)
Others	
Epiretinal Membrane	3 (0.95)
Macular pucker	2 (0.63)
Retinal detachment	1 (0.32)
Vitreous Hemorrhage	2 (0.63)
Asteroid hyalosis	1 (0.32)

A patient can have more than one finding; for example, cataract, mild NPDR, and epiretinal membrane

*Legend*: *NPDR* Non-proliferative diabetic retinopathy

In the bivariate analysis, follow-up was associated with sex, race, being married, being imaged at a UPMC site, smoking status, severity of DR, having non-gradable images, and having incidental findings including suspected glaucoma, AMD, hypertensive retinopathy, BRVO, and other diagnosis (Table 6).

**Table 6:** Sociodemographic and clinical characteristics of follow-up eye care after a referral to an ophthalmologist (row percentages, *n* = 2,493)

Characteristics	Follow-up status		*P*-value
	**Not followed up (*n* = 1706)**	**Followed up (*n* = 770)**	
Age, mean (SD)	60.20 (12.80)	60.32 (13.14)	0.84^†^
Sex, n (%)			0.04^*^
Female	802 (67.17)	392 (32.83)	
Male	921 (70.90)	378 (29.10)	
Race, n (%)			0.003^*^
Black	711 (66.14)	364 (33.86)	
Others	112 (78.18)	36 (21.82)	
White	883 (70.47)	370 (29.53)	
Marital status, n (%)			< 0.0001^*^
Married	629 (66.28)	320 (33.72)	
Non-married	1,024 (69.71)	445 (30.29)	
Insurance type, n (%)			0.35^*^
Commercial	385 (68.02)	181 (31.98)	
Medicaid	318 (70.51)	133 (29.49)	
Medicare	666 (68.84)	300 (31.06)	
Dual	179 (66.79)	89 (33.21)	
Veterans Affairs	5 (45.45)	6 (54.55)	
Uninsured	37 (71.15)	15 (28.85)	
Quartile of poverty, n (%)			0.05^*^
< 10% (Q1)	865 (68.43)	399 (31.57)	
10–19.9% (Q2)	619 (69.55)	271 (30.45)	
20–29.9% (Q3)	124 (73.37)	45 (26.63)	
30–39.9% (Q4)	84 (62.69)	50 (37.31)	
Clinic Administration, n (%)			< 0.001^*^
UPMC	1.314 (64.38)	727 (35.62)	
FQHC	183 (93.85)	12 (6.15)	
Free clinics	47 (77.05)	14 (22.95)	
Healthcare gaps	178 (91.28)	17 (8.72)	
BMI, median (IQR)	32.07 (27.36–37.38)	32.02 (27.52–37.45)	0.64^‡^
HbA1C, median (IQR)	7.6 (6.5–9.3)	7.3 (6.45–8.75)	0.37^‡^
Smoking status, n (%)			< 0.0001^*^
Former smoker	560 (66.99)	276 (33.01)	
Never smoked	642 (65.58)	337 (34.42)	
Smoker	419 (73.12)	154 (26.88)	
Type of diabetes, n (%)			0.18^*^
Type 1	55 (64.71)	30 (35.29)	
Type 2	1,544 (68.90)	697 (31.10)	
Degree of DR, n (%)			< 0.0001^*^
Mild	82 (70.69)	34 (29.31)	
Moderate	170 (62.27)	103 (37.73)	
Severe	29 (46.03)	34 (53.97)	
Proliferative	25 (51.02)	24 (48.98)	
Macular exudate	24 (53.33)	21 (46.67)	
No DR	706 (69.63)	308 (30.37)	
Other findings, n (%)			
Non-gradable images	641 (75.32)	210 (24.68)	< 0.0001^*^
Glaucoma suspicion	380 (62.50)	228 (37.50)	< 0.0001^*^
AMD	100 (68.97)	45 (31.03)	0.01^*^
Hypertensive retinopathy	67 (70.53)	28 (29.47)	0.01^*^
BRVO	32 (53.33)	28 (46.67)	0.001^*^
Other findings	73 (64.60)	40 (35.40)	0.005^*^

*Legend*: *SD *Standard deviation, *IQR* Interquartile range, ^†^. t-test, ^‡^. Wilcoxon test, ^*^. chi-square test, *DR *Diabetic retinopathy, *VTDR *Vision-threatening diabetic retinopathy, *AMD* Age-related macular generation, *BRVO* Branch retinal vein occlusion

The multilevel logistic regression model for associated factors with follow-ups clustered by site and patient, show no associations with sex, race, insurance, socioeconomic status, smoking status, a diagnosis of glaucoma suspicion, or the time between the exam and report (*p* > 0.05). We found that older people were more likely to follow up, with an increase in this likelihood as age categories increased. However, only one age group was significantly associated with follow-up after adjustment. Patients between 66 and 75 years were 77% more likely to follow-up than those between 18 and 45 (OR = 1.77, 95%CI = 1.10–2.85). Married patients were more likely to follow up with a 38% higher probability of seeking care than those who were single (OR = 1.38, 95%CI = 1.03–1.84). Finally, a diagnosis of severe DR was associated with the highest likelihood of follow-up (OR = 2.69, 95%CI = 1.30–5.53; Table 7). The ICC for sites was 11.83% (standard error, SE = 4.93%) and 20.62% (SE = 12.82%) for patients; therefore, the clusters explain a relevant part of the model variance.

**Table 7:** Factors associated with following-up after a recommendation of an in-person eye exam from the POCDREP

Characteristic	Unadjusted OR	95% CI	*P*-value	Adjusted OR	95%CI	*P*-value
Age groups (18–45)	(ref)					
46–55	1.15	0.81–1.63	0.45	1.14	0.75–1.74	0.54
56–65	1.19	0.85–1.66	0.32	1.39	0.91–2.12	0.13
66–75	1.17	0.82–1.66	0.38	1.77	1.10–2.85	0.02
76–97	1.18	0.78–1.78	0.42	1.45	0.80–2.62	0.21
Sex (Male)	(ref)					
Female	1.13	0.92–1.37	0.24	1.23	0.95–1.61	0.12
Race (Other)	(ref)					
Black	0.93	0.53–1.65	0.81	0.95	0.42–2.15	0.91
White	0.88	0.50–1.57	0.67	0.66	0.29–1.47	0.31
Married (Single)	(ref)					
Married	1.33	1.07–1.64	0.009	1.38	1.03–1.84	0.03
Insurance (Other)	(ref)					
Commercial	0.98	0.78–1.24	0.89	1.02	0.75–1.40	0.88
Socioeconomic status (0–9.9% Q1)	(ref)					
10–19.9% (Q2)	0.88	0.69–1.11	0.28	0.92	0.68–1.26	0.62
20–29.9% (Q3)	0.76	0.48–1.18	0.22	0.67	0.38–1.20	0.18
30–39.9% (Q4)	1.00	0.64–1.56	0.99	0.74	0.39–1.38	0.34
Smoking status (Never)	(ref)					
Former Smoker	0.89	0.71–1.12	0.33	0.98	0.73–1.32	0.91
Smoker	0.66	0.50–0.86	0.002	0.70	0.49–1.01	0.06
DR level (No DR)	(ref)					
Mild	1.09	0.67–1.75	0.73	1.16	0.68–1.98	0.59
Moderate	1.21	0.88–1.66	0.25	1.28	0.86–1.90	0.22
Severe	2.47	1.33–4.57	0.004	2.69	1.30–5.53	0.007
Proliferative	1.55	0.81–2.96	0.19	1.75	0.83–3.73	0.14
Macular exudate	1.54	0.79–3.01	0.20	1.58	0.75–3.35	0.23
Glaucoma suspect (No)	(ref)					
Yes	0.96	0.76–1.23	0.76	1.02	0.73–1.41	0.91
Time between exam and report (0 days)	(ref)					
1 day	0.88	0.51–1.52	0.65	0.72	0.35–1.48	0.37
2–3 days	0.90	0.53–1.54	0.71	1.04	0.55–1.98	0.89
4–6 days	0.66	0.37–1.21	0.18	0.73	0.34–1.56	0.42
> 6 days	0.86	0.55–1.35	0.51	0.84	0.46–1.52	0.56

*Legend*: *ref*: category of reference, *POCDREP* Point-of-Care Diabetic Retinopathy Examination Program, *DR* Diabetic Retinopathy

## Discussion

In this study, we report the outcomes of the POCDREP, including patients’ characteristics, diagnoses made, follow-up rates, results of in-person follow-ups in indeterminate results, and associated factors with follow-up. Previous studies have reported on the outcomes of similar DR photographic examination efforts in the US and abroad [29, 38]. However, this is among the few studies describing follow-ups and their associated factors, and it is the study with the largest sample of patients requiring follow-up [16, 31–33, 45, 46]. We also describe findings at follow-up with eye care after ungradable imaging, from which there is scarce evidence [38].

Our study demonstrates that the program was helpful for detection, diagnosis, and referral, potentially preventing adverse outcomes. Compared to other programs, ours was implemented in an extensive and diverse range of primary care practices within the Pittsburgh area for the insured and uninsured/indigent population.

Other studies on point-of-care DRE in the US only report on the results of a single primary care practice [28], specific populations like Latinos or American Indian/Alaskan [23], or clinics within the same healthcare insurance system [47]. Each of these studies focused on the feasibility of the program [23, 28, 47, 48], but little about follow-up after a positive or inconclusive exam. Studies typically recommend an eye care assessment, but do not provide information about whether the assessment was conducted, nor the results obtained [23, 45, 47, 49]. Lack of information about follow-up outcomes of patients with indeterminate exams is concerning, as our research indicates these individuals may have potential ocular issues requiring further assessment and intervention.

During the four years of data collection, the program examined 6,245 PwDM. Though the study’s patients were predominantly White (representatives of Pittsburgh’s demographics); however, 35.1% of this study´s patients identified as racial/ethnic minorities, 5% of patients resided in areas of poverty, and 41% were enrolled in Medicaid, VA, or were uninsured. Low socioeconomic status and racial/ethnic minorities are associated with higher rates of DR and lower rates examinations [50–52]. Likely, the program is accomplishing its aim to reduce the barriers to eye care faced by high-risk individuals in the city and its surrounding areas due to the variety of settings where the examination occurs [53].

Out of 727 providers, only 14.6% (106) ordered half of the total examinations. This concentration among a minority of providers limits the program’s reach and effectiveness. Resistance from some providers, staff, and patients who question the DRE’s effectiveness or see it as a replacement for in-person exams may contribute to this concentration [54]. Another possible cause is the fixed location of most cameras, primary care sites without cameras refer patients to sites participating in the POCDREP for DRE, resulting in the orders being attributed to the latter sites. However, this seems to be the case on a small proportion of exams as the program referral system begin to work late in 2018. It is essential to ensure that primary care physicians have adequate knowledge about DREs to establish a coordinated referral system and ensure timely examination [55].

We believe that it is possible to increase DRE utilization through outreach and education initiatives, as well as incentive such as bonuses for number of DRE conducted on-site. Additionally, offering examinations for the family and friends of PwDM in locations with available cameras can help promote the importance of DR detection and prevention. Studies show that DR diagnosis via traditional fundoscopy was found suboptimal at primary care cites [46]. Thus, DREs, which are less invasive and more cost-effective, can offer a better option for detecting DR in eligible patients.

The POCDREP achieved a high rate of interpretable images (81.1%) and detected a similar DR (12.6%) and VTDR (6.3%) prevalence as other programs in which gradable images range from 67–89% [27, 49], DR prevalence between 15–33%, and VTDR between 5–7% [15, 16, 20–23, 28, 29, 37]—underscoring the program’s effectiveness in diagnosing and screening ocular conditions and its reliable implementation. Nonetheless, we observed a higher incidence of ungradable images in the left eyes (*p* < 0.05). This outcome is likely due to the right eye being photographed first with a flash of light that contracts both pupils. Swift transitions between eyes during imaging are a probable factor contributing to this issue. Further quality improvement efforts should attempt to address this situation.

Most images taken on the POCDREP were interpreted within 24 h; this rapid turnaround time is essential for ensuring prompt referral to ophthalmologists and the timely initiation of treatment, which can help prevent vision loss and other complications associated with DR [56]. We hypothesize that the shorter the time between the exam and the report, the higher the odds for follow-up. Nevertheless, in the multivariate model, this variable was not significant, which could be due to unadjusted counfunding factors. Further research is required to identify the effect in follow-ups of time since the report and the provision of the information to the patient.

While conducting DRE, we found that 23% of exams were positive for pathologies other than DR, including suspected glaucoma, AMD, hypertensive retinopathy, and branch retinal occlusion. Other studies that also noted these ocular findings during DRE found rates ranging from ~ 25–44% [21, 28, 57]. Though these are secondary findings within the program, they indicate the program’s benefit as an effective method for detecting potentially vision-threatening ocular conditions other than DR that can be identified and, in most cases, treated. Notably, a small proportion of gradable exams identified various abnormal retinal findings, such as epiretinal membranes, choroidal nevus, and chorioretinal and macular scars (1.9%). These rates are consistent with similar programs, which report finding such abnormalities in approximately 10–11% of cases [28, 57]. These findings require further eye specialist evaluation because of their associations with serious ocular conditions, including retinal tear/detachment, melanoma, and infection [58–60].

With approximately a third of exams being indeterminate, these patients were instructed to have a follow-up appointment with an ophthalmologist. Within a year after the initial DRE, 33.8% had had a consultation, of which nearly two-thirds (63.5%) had visually significant findings such as cataracts and glaucoma, and 18.4% had VTDR, indicating a high detection rate of findings even with indeterminate exams [61]. Other similar programs do not describe follow-up visits to eye care in patients with indeterminate results [45, 47, 49]. Our results are vital to demonstrate that PwDM with ungradable exams are likely to have a vision-threatening condition. It is crucial to put more effort into follow-up of patients with non-gradable/indeterminate exams. This can be achieved by recommending follow-up appointments or considering a re-uptake of the retinal images after pupil dilation to have clear evidence of disease and expedite the referral process.

Follow-up rates six months after DRE were low (30.9%). Compared with other programs in the United States, the referral adherence rate of our program falls within the range of 10–62% [27, 28]. To assist patients without established eye care, the POCDREP provides information about findings and scheduling services for follow-up appointments. Improving adherence to follow-up appointments can present challenges due to disparities in eye care provision [62]. Interventions that include personalized approaches and community-based programs have demonstrated effectiveness in improving access to eye care and increasing patient adherence to continuity of care recommendations [63].

In the POCDREP, we have implemented a personalized approach to improve adherence to follow-up recommendations. Patients with a positive or indeterminate exam are contacted and informed about the exam results so they can be assisted with scheduling the follow-up appointment. Our personalized approach included at least three phone calls to attempt to reach the patient, voicemail messages if there was no response, and a letter informing the patient of their results and the requirement of an appointment with an eye doctor. A similar personalized intervention trial (personalized phone call, letter, appointment reminder, and phone call reminder) showed higher adherence to recommendations to schedule and visit the eye center for DRE, compared with usual care and automated interventions [64]. Further research is required to identify barriers to follow-ups in the POCDREP and interventions to improve follow-up rates.

In the subgroup of patients recommended for follow-up for VTDR, 42.23% followed up, comparable with the 51–76% rates achieved by similar programs [29, 31]. We report that patients with VTDR were followed up more quickly compared with when other diagnoses triggered the referral. As far as we know, this has not been reported in the literature.

We found that older (> 65 years) patients are more likely to comply with follow-up, which supports the results of previous studies [9–11, 30, 65, 66]. Being married was also associated with following up with eye care. It is well known that having a social and familial network helps with better health outcomes [67].

The strongest factor associated with adhering to recommendations for follow-up appointments was having a more severe form of DR, indicating a threat to vision [68]. The report of a severe disease may motivate the patient to get treatment, as losing sight is one of the most frightening situations for a person [69, 70].

There are documented racial disparities in eye care provision among working adults in the US [30, 51]. Other studies have found associations between income and lower adherence rates [30, 71]. However, we did not find associations with follow-up status and race or a surrogate of income using ZIP codes. Our study included a large proportion of minorities and people with some access to healthcare, as most of them were examined in a clinic (only 2.26% of patients had a DRE in outreach activities). Patients required to follow-up had to be examined, therefore some barriers for screening will not be relevant for follow-up. Nevertheless, further research is required to understand the low follow-up rates of our program.

### Future directions

This study can point out to the development of interventions to prevent patients not following-up: such as targeted communication strategies based on age groups, community programs to reach out single patients or without a social support network, and intensified follow-up protocols and patient education about the effects of different levels of retinopathy [72].

For those less likely to follow up, such as single individuals or those with milder forms of DR, community health interventions could be beneficial. These might include support groups, community health worker outreach, and educational seminars to highlight the risks of not pursuing follow-up care [72].

Within healthcare systems, policy changes could be advocated to integrate automatic follow-up appointments into diabetic retinopathy screening protocols, particularly for patients with severe DR. This would ensure comprehensive patient management and prevent any patients from being overlooked.

The lack of significant associations with expected factors such as socioeconomic status, race, and system-level factors like the time between imaging and reporting suggests further research to understand the nuanced barriers to follow-up care. Future prospective studies should focus on follow-up assessments and interventions to improve adherence. Potential strategies include implementing phone call protocols, providing incentives for continued participation in the program, offering personalized care, establishing support groups, and delivering educational assistance.

### Limitations

The main limitation of our study is that the covariables used in the analysis of associated factors were limited to those coming from the automatic monthly EMR data reports and are retrospective in nature. This introduces some inherent limitations, such as selection bias, as the cohort was selected from existing records. However, we included all records within the study period. Data quality and completeness pose challenges, as some PwDM were lost to follow-up. Additionally, there may be uncontrolled confounding variables that were not accounted for. The included variables provide some insight into the patient characteristics; however, we were primarily limited to socioeconomic factors rather than variables describing the patient-healthcare system relation. Further research should include variables that could be modifiable factors to prevent loss to follow-ups.

## Conclusion

The POCDREP serves a wide variety of clinical settings covering both insured and uninsured/indigent populations. The six-month follow-up rate was found to be suboptimal. Patients with indeterminate exam are at risk of vision-threatening conditions, thus highlighting the importance of addressing this outcome in DRE programs. Future efforts should prioritize continuous education, incentive programs, and personalized approaches for physicians and patients to increase awareness, camera use, and adherence to DRE and follow-up recommendations. We described associated factors to follow-up as being older age (66–75 years of age), being married, and severe DR. Further research is required to identify modifiable factors to adherence for DRE recommendations that lead to the design of interventions.

## Data Availability

Data is provided within the manuscript or supplementary information files, further datasets used and/or analysed during the current study are available from the corresponding author on reasonable request.

## Abbreviations

POCDREP: Point-of-care Diabetic Retinopathy Examination Program
UPMC: University of Pittsburgh Medical Center
DR: Diabetic Retinopathy
DRE: Diabetic Retinopathy Examination
EMRs: Electronic Medical Records
AAO: American Academy of Ophthalmology
VA: Veterans Affairs
AMD: Age-Related Macular Degeneration
VTDR: Vision-Threatening Diabetic Retinopathy
BRVO: Branch Retinal Vein Occlusion
ER: Emergency Room
PCP: Primary Care Physician
FQHC: Federally Qualified Health Center
